# How community sport programs may improve the health of vulnerable population groups: a program theory

**DOI:** 10.1186/s12939-020-01177-5

**Published:** 2020-05-24

**Authors:** Karen Van der Veken, Emelien Lauwerier, Sara J. Willems

**Affiliations:** 1 Department of Public Health & Primary Care, Ghent University, Corneel Heymanslaan 10, 9000 Ghent, Belgium; 2 Department of Experimental-Clinical & Health Psychology, Ghent University, Ghent, Belgium

**Keywords:** Theory-building, Community sport, Socially vulnerable, Health promotion

## Abstract

**Background:**

Groups at risk of exclusion from society appear to have a lower health status and more health-related problems. Prevention efforts in these groups are not always successful, and new ways have to be sought by which health messages can be delivered. Many agree on low-threshold sport activities, also called ‘community sports’, to be a powerful tool to target socially vulnerable groups. Until now, it has not been investigated how and when such sport initiatives may be able to impact health outcomes in socially vulnerable populations. This study aims at developing a program theory that clarifies the mechanisms and necessary conditions for sport programs to be effective in health promotion. Such a program theory may constitute a backbone for developing health promotion initiatives within a sport for development setting.

**Methods:**

We developed a program theory using a realist research design. We build on an extensive data set consisting of the insights of key stakeholders and participants of various community sport organizations at the one hand, and on relevant theoretical frameworks at the other hand. Data were collected through participatory observations of soccer trainings and related group activities, interviews with key stakeholders and participants, document analysis and two focus groups with stakeholders from associated social partnership organizations.

**Results:**

The health promoting effect of community sport on socially vulnerable groups seems not to result from an improved physical condition or sport-technical skills as such, but from processes of experiential learning among peers, incremental responsibility-taking and reflexivity. On the condition that participants feel safe, are stimulated to reflect and enabled to become actor of themselves and their situation, these processes are likely to lead to increased self-esteem, self-efficacy and motivation to set and pursue personal (health) goals. The key-influencing factor in these processes is the coach, who therefore needs to be adequately skilled in, for example, social vulnerability, motivational coaching and group dynamics.

**Conclusions:**

The program theory developed in this study offers insights in the mechanisms proper to, and necessary conditions for community sport to be a lever for health promotion in socially vulnerable groups. Motivational processes at individual level and group connectivity are at the basis of personal health goal-setting. One of the necessary conditions is that these processes are guided by community sport coaches skilled in the meaning and impact of social exclusion, and capable of connecting with the target group.

## Background

Social exclusion is probably the most accurately defined as “the lack or denial of resources, rights, goods and services, and the inability to participate in the normal relationships and activities, available to the majority of people in a society, whether in economic, social, cultural or political arenas” [1].

It has both a direct effect on the physical and mental health of the socially excluded [2–5], and many indirect effects. Since social exclusion touches to all aspects of people’s lives, it complicates the implementation of preventive strategies for intervention. For example, socially excluded people do not participate as often or as thoroughly in contexts that are used as a setting for health promotion, such as work, school or mass-media campaigns [6, 7]. Furthermore, messages do not always reach the target group because of feelings of isolation, a lack of relatedness, an overall sense of hopelessness or frustrations with policy measures [8]. Socially vulnerable groups might also have other priorities or concerns than the subject of the message sent out to them. Even when the message has arrived and awareness is there, several obstacles remain for socially vulnerable individuals to (be able to) undertake action to improve one’s quality of life [3, 7]. A working single mother, for example, may be well aware of the importance of physical activity, both for her children and for herself. Yet between awareness and action are many constraints, such as geographical (too far to go by foot), financial (too expensive to take public transport, to buy the required outfit…) and cultural ones (elite sport club, communication through social media, training 4 times a week required…). Moreover, fresh and healthy food may be a concurrent priority, as is time and attention to follow up on school progress of the kids, and so forth.

Since social exclusion is a complex and multi-dimensional process [1], promoting health and well-being among socially vulnerable groups is a complex social intervention requiring a multifaceted understanding and policy response, applying principles of proportionate universalism. Population-wide universal interventions (through schools, sport and leisure clubs, employment offices, etc.) should be completed with specific interventions targeting vulnerable population groups, varying in both the intensity of the intervention and the methods used so that they address the specific living conditions in which vulnerable groups live and work [7]. Sport, among others because its attractiveness among youth, may be a context of interest in which such multifocal response can start from, on the condition that it enables to reach the ‘hard-to-reach’. Participation in sport is increasingly considered an effective instrument to enhance the ability of the most vulnerable in society to cope with adversity [9–13]. For children and adolescents, sport has shown to be related to reduced anxiety, higher self-efficacy, self-confidence and social benefits such as higher investment in meaningful relationships and feelings of connectedness [14]. However, since social exclusion also touches to the domain of leisure and sport, the classical sport club is not the most effective setting for using sport as a tool for health promotion [15]. Because of its potential to overcome these barriers to sport participation, ‘community sport’ has been studied from the late 1990s onwards [16–21]. Community sport activities are low threshold and financially accessible, and organised locally, in specific – often urban – neighbourhoods. The activities are not usually high level or competitive in nature. The above aspects make the community sport setting a fitted context for meeting like-minded people in a safe and accessible manner, and potentially a powerful tool to reach socially disadvantaged groups. Consequently, community sport has earned a place on the global, European and local social policy agendas [9–13], and is increasingly being integrated, particularly in the developing world or in divided societies, in community development strategies to contribute to reconciliation and peace, and to pursue the Millennium Development Goals (http://www.un.org/ millenniumgoals/). Sport-for-development, as it is called in this context, has been, among others, implemented to tackle discrimination and encourage respect; bridge social, cultural and ethnic divides; combat non-communicable diseases and HIV/AIDS; contribute to gender equality; and healing the psychological wounds of traumatized victims of natural or human-made disaster [22].

Although many successful community sport practices exist and their – not always systematic – effects on the well-being of socially excluded groups have been documented [16, 20, 23, 24], a generalizable program theory is still missing. The aim of this study is to develop a program theory on how, in which circumstances and to what extent community sport may improve socially vulnerable participants’ health and well-being. We look for the key mechanisms through which community sport addresses individual’s resilience and positively impacts health, and for determining context factors, some of which are necessary conditions, others mere facilitating. Such program theory informs stakeholders on what working elements should be triggered and what context needs to be in place in order for a health promotion program using sport as a lever to be successful. In times where many projects with social affinity lack long term budgetary visibility, an overview of factors facilitating a successful outcome of the project, is likely to serve both stakeholders and policy makers.

## Methods

### Study design

This study is part of the CATCH research project, a four-year (2016–2019) transdisciplinary research project designed to identify the underlying social mechanisms of community sport that relate to personal development, health and social cohesion, and enabling conditions (context factors at micro, meso and macro level) for these social mechanisms to function. This paper focuses on the findings regarding health. The findings related to personal development and social cohesion are reported elsewhere [12, 25].

Our research question is embedded in a realist research design. Realist evaluation [26] aims to identify the hidden causal forces behind empirically observable patterns or changes in those patterns [27]. This is done through ‘retroduction’: going back from observed patterns and looking below the surface for what might have produced them [28, 29]. Hereby, realist studies focus on context and necessary conditions for social mechanisms to be generated, which makes it a useful approach for studying complex social issues such as health [30]. More concretely, we used steps from the classical grounded theory approach (GTA) to build theory from case studies in an overall realist inquiry study design [26, 31, 32]. We chose for this approach because of the appreciation of realism as a ‘logic of inquiry that generates distinctive research strategies and designs, and then utilizes available research methods and techniques within these’ [33]. In this study, we started from the empirical outcome (an important commonality between GTA and realist evaluation), tracing processes backwards to study the question ‘what is it about community sport that works for socially vulnerable populations, why is that and under which circumstances?’ [33]. The output is a program theory (PT), developed at a mid-level range of abstraction, i.e. a theory concrete enough to test yet generalizable to different contexts, therefore called a ‘Middle-Range Theory’ (MRT) [34]. This program theory clarifies why, how and in which circumstances community sport can promote health (respectively referring to mechanisms and influencing context factors of improved health outcomes).

### Data collection

Data were collected iteratively. First, observations (January–April 2016) were conducted in three local football teams consisting of people in socially vulnerable situation, located in three Belgian cities of different sizes. One hundred and nine hours of participatory and non-participatory observation during trainings, leisure moments, team-building activities, staff meetings, and national and local tournaments provided insights into the organization of the teams, their partnerships, the participants they reach and the activities they offer. These insights were recorded in field notes immediately after each activity. Additionally, in-depth interviews were conducted (cf. Additional file 1) with 22 coordinators and social partners of different community sport initiatives, and with seven participants from the three teams that had been observed in the first round of data collection (May–November 2016). There were two selection criteria for the (stakeholder / participant) interviewees: 1) diversity, to ensure respondents of different age, gender, ethnic background, occupation and partner organization; and 2) the respondents’ knowledge of the daily functioning of the team. To ensure those criteria were met, interviewees were chosen in collaboration with project coordinators. All interviews were semi-structured, using an interview guide based on the observations. The research team and experts discussed the guide and revised it after two test interviews in order to reveal more easily the key mechanisms of community sport and facilitating context factors. The interviewers started by asking about any health-related effects respondents experience through community sport, and then asked how respondents think these effects come about and which context factors are necessary for allowing these effects to occur. Where possible, interesting data from previous interviews were discussed and refined in later interviews. Finally, two focus groups (*N* = 6 and *N* = 7) were organized (February 2017) with coordinators, coaches and partners from various community sport organizations, in order to discuss and validate or adapt the initial program theory presented (cf. Additional file 2). The study team then identified and explored gaps, contradictions and uncertainties in the data from the interviews and observations. The focus group guide was refined and validated through meetings with international experts and within the research team. Interviews and focus groups were audio-recorded and transcribed verbatim.

Ethical approval for this multiple-case study was obtained from the Ethics Committee of the Ghent University Hospital (EC registration number: B670201628570).

### Data analysis

Four analytical steps were taken after data collection: 1) open coding of data (identifying the sensitizing concepts); 2) axial coding of data (creating explanatory accounts); 3) selective coding of data (consolidating accounts); and 4) structuring consolidated accounts in a program theory.

#### Step 1: identifying sensitizing concepts

Data from observations and interview data were coded inductively in nodes in NVivo 11. We used the following criteria to select a core variable during the coding process: centrality, frequency, relevance, grab and variability [35]. This means that the core variables, further described as ‘sensitizing concepts,’ were of central concern for the participants in the study, appeared frequently and with a stable pattern in the data, related meaningfully to the concepts’ different variables, were imaginary and explanatory, and could be discovered in other substantive areas beyond the area from where the concepts emerged [36].

#### Step 2: creating explanatory accounts

Working ‘backwards’ from outcomes, the sensitizing concepts were labelled as an outcome (O), a context factor (C) or a mechanism (M) [37]. NVivo’s coding queries were then used to find overlaps between outcomes and mechanism or context categories. These coding queries identified which fragments of interviews overlapped and which sensitizing concepts were coded to these fragments. Out of all overlapping fragments, recurring outcomes (O), mechanisms (M) and context (C) factors were identified, and reformulated (where possible) as ‘*if* … *then* … *because*’- statements as such obtaining ‘explanatory accounts’ [38]. ‘If’ is followed by a context factor, ‘then’ by an outcome on initial, intermediate and/or distal level and ‘because’ precedes what the study team could extract from the data as main reasoning on why and how the concerned outcome occurred in that specific context (mechanism). All explanatory accounts (*n* = 432) were listed in a table, together with the source of the statement.

#### Step 3: consolidating accounts

Two researchers (KV, EL) reviewed and discussed the inter-relationships and overlaps between explanatory accounts in order to decide which account to import directly into the consolidated explanatory accounts table, which account to merge with another and which account to reject. Following Pearson et al.’s strategy, this discussion was guided by the following questions: Is this account novel? If not: does this account challenge the explanations made in related accounts? Does this account add important refinements to the understanding of contexts, mechanisms, or outcomes made in related accounts? [38]. Whenever inconsistencies emerged in this process, a third reviewer (SW) was consulted. The explanatory accounts where consolidated in 16 dense accounts that were presented in the form of an initial program theory (cf. Additional file 2) to stakeholders in two focus groups for further discussion and potential consolidation. The whole of the consolidation process lasted for several months and was characterized by multiple feedback loops, emergence and non-linearity. In the end, four consolidated accounts in the form of CMO configurations remained (cf. Results).

#### Step 4: from consolidated accounts to program theory

In this step, the four CMO configurations were linked to one another, taking into account that the outcome of one CMO configuration might represent the necessary context to decline the central mechanism of another CMO configuration, and vice versa. These associations, as well as the supposed proximity and chronology in the relation among the CMO configurations, were presented in a visual or schematic diagram in the form of arrows, circles with common parts, etc. Three researchers familiar with the data discussed these schemes and figures with the social users (i.e. all community sport organizations within the network) of the CATCH project. While discussing on how the sensitizing concepts and consolidated accounts fitted together in a model, the process of cross-pollination in qualitative research became clear [39]: although the researchers tried to analyze and interpret the data grounded in their specific contexts, the theory that was developed from this analysis inevitably showed some resemblance to existing theories and social sciences concepts, e.g. the social cognitive theory and the self-determination theory [40, 41]. This influenced the process of naming the sensitizing concepts and key mechanisms, and of developing hypotheses on the relations between variables.

## Results

**Fig. 1:**
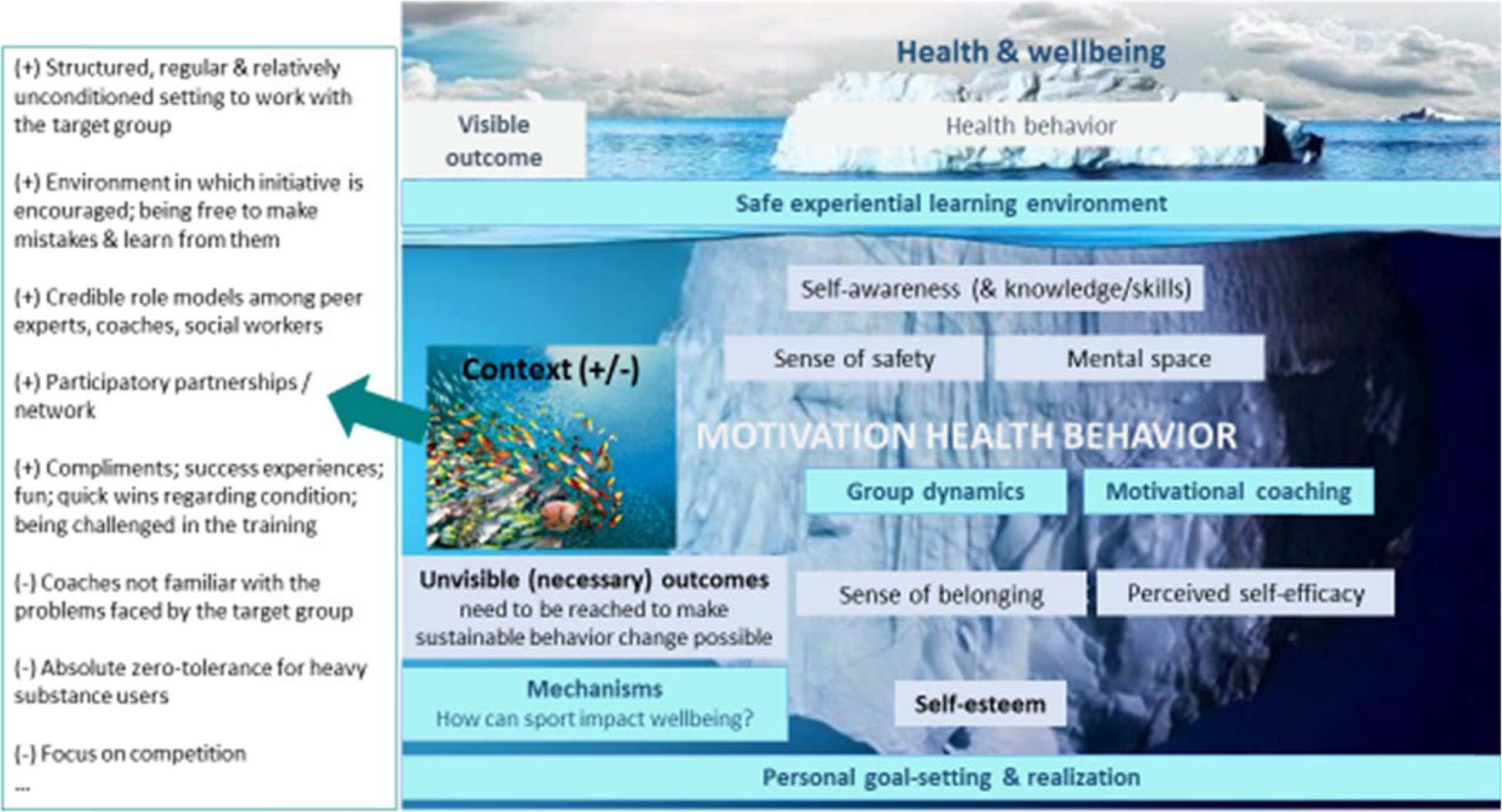
Community sport as lever for health and well-being: a program theory

First, the four consolidated accounts (CMO configurations) that resulted from the third analytical step (cf. Table 1) are described. Outcomes are split in initial outcomes (iO), intermediate outcomes (IO) and distant outcomes (DO). Second, it is explicated how these CMO configurations are linked together in an overall program theory (Fig. 1).

**Table 1:** Examples of verbatim & facilitating context factors inspiring the CMO configurations

CMO configuration	Examples verbatim used for CMO configuration	Facilitating context factors
**A safe haven to start from (CMO1)**	*You notice that, once people feel at home and safe, there are some things that come up on which we, hopefully, can build further. (R16)* *Young people radicalize because they have nothing to do, because they’re receptive for those… Give them a structure, give them a goal, give them something to be proud of (…) Make sure it stays accessible and that the offer is broad, including other leisure and cultural activities. It does not have to be about sport. (R3)* *Instead of being in class and not understanding half of things, feeling depressed (…). Some have not seen their dad or mom in 3 years. Well, they’re preoccupied with all that. And sporting is then … to not have to be preoccupied with that for a while, and simultaneously, because of the accessibility of our activities, still feeling that there is space to talk about that. (FG1f)*	▪ Community sport coaches naming and personally greeting all participants ▪ Coaches inviting, though not obliging, participants to discuss problems and/or feelings ▪ Coaches practicing a signal and referral function and intervening when they sense a participant does not feel well or behaves inappropriately ▪ Coaches creating partnerships with other community (social or educational) workers so that learning is expanded outside the sports activities themselves.
**Improved self-efficacy through motivational coaching (CMO2)**	*Now, there is no more ranking (…). And we often win the ‘fair play cup’, so it shows that this motivates the players and that they join this idea. Yeah, it makes sure that everyone feels good within the team. When there is no focus on winning or if this is not the main goal, a player that is a little less skilled will also get the confidence****.****(R16)* *What we find really important is positive coaching, starting from people’s strength. Those are people that fail very often, and if you as a coach, during a football training also start to talk about the things they don’t do well, then it goes wrong. We name what they do well, even if that is a very little thing. (R3)*	▪ Explicitly appreciating the fact that participants who experience the most thresholds for physical activity, made it to training (as such motivating them to come again) ▪ Regularly pointing to positive behavior or reactions of participants that they themselves may be unaware of, and stimulating participants to compliment others, and themselves ▪ Appreciating effort over result and avoiding to compare participants with one another.
**Sense of belonging and self-esteem through constructive group dynamics (CMO3)**	*I feel useful and valued, yes. I feel useful because I can play in the [soccer team for socially vulnerable participants linked to a well-known First Division soccer team] and I feel valued, yes, the other players value me because I play there and because I sometimes help people (R28)* *(Asked about what it is about the homeless soccer team that ‘works’)* *I think… to belong. That there are no prerequisites, that you are always welcome. If you have never known that, it is a very strong thing to experience… that this is allowed and that you can be yourself. (R22)* *Now we use elements that focus on connecting: using games, running in group, starting and closing the training in group. (…). And in the beginning they asked for matches, but after some time that changed and then you really feel that it has a big impact on the group, by working differently with them. (R13)* *Because you have social contact again, you have more social contact actually. In the past I did not leave the house, and just sat in my room every day. And then I just started to take some steps. First […], then […], the football, then the youth movement. (R29)*	▪ Greeting (and naming) every participant before the start of an activity ▪ Actively introducing new participants and using the opportunity to enlarge all participants’ acquaintance, e.g. through games that allow to get to know one another during the sport activity ▪ Integrating a group enhancing activity in every sports activity (in case of individual sport, this could be a warm-up in group) ▪ Ensuring an optimal role distribution in the group in the sense that all participants have a specific role to play in the activity and that roles are shifted (by the coach or an appointed team leader) from time to time ▪ Guarding constructive interaction (communication, feedback) with and between participants at all times ▪ Stimulating participants to establish a common goal and motivating them to pursue it ▪ Making use of role models to reinforce positive group feelings, e.g. by linking the team to a Premier League team ▪ Organizing activities outside of the sports trainings, e.g. tournaments (eating, travelling, warming up... together) or participation in social events
**Mentoring participants in personal health goal-setting (CMO4)**	*Our training is a location where people can meet, and where we can build a positive relation with people, to then work on several life domains on other moments. (…) We work very broadly: housing, administration, psyche, relations, addiction… But we work around these themes at the moment that people come up with something. They determine the agenda; we try not to push too much in one or another direction. (R14)* *We come off from the traditional welfare context and actually… create an environment in which we can work with the people without them… having the feeling that is forced upon them. They want it themselves. It happens upon their request. (R3)*	▪ Presence of a (realistic, achievable) technical challenge in the training ▪ Existence of a clear group goal to which participants can link their personal goals (e.g. participating in a tournament) ▪ Adapted exercises for participants with less developed sportive skills (i.e. tailoring) without neglecting the more advanced players or the group dynamics ▪ Opportunities to take initiative and to grow in responsibility or engagement (e.g. making players who grew in confidence and in sport-technical skills responsible for the sport gear or an informal deputy trainer (positively coaching) his/her peers) ▪ Coaches providing participants with an individual training schedule that is feasible and matched to the condition level and preferences of the participants (individualization, tailoring) ▪ An adapted environment to make healthy choices more easy (e.g. replacing the candy machine by a healthier offer; foreseeing a source of drinking water and setting clear rules (e.g.: no smoking on the sports field) ▪ Coaches with knowledge of substance use and how to deal with them (who, e.g., support users without judging them, persuade participants to at least not be secretive about their use and maybe talk to them about it) ▪ Partnerships for improved exchange of information and more fluent transfer to social partners who can assistant participants in realizing their personal health goals

### A safe haven to start from (CMO1)

***If****community sport activities are predictable, structured and relatively unconditioned (C),****then****the participants experience a sense of safety and acceptance (iO) which motivates them to be engaged in community sport (IO) and have trust in peers and coaches (IO)****because****they perceive community sport as a setting in which they can ignore or even unload their emotional baggage, have fun, and be themselves among trustworthy peers (M).*

When participants experience predictability in daily life – i.e. they can count on things (e.g. a sport training) and on people to be as expected – it makes them more secure in their interactions and allows them to relax, as such creating contributing to what we have labeled “mental space” in the program theory: the space created in one’s head when one is temporarily released from daily responsibilities and heavy emotional luggage. This liberated mental space can be used to be fully present in the moment and work on one’s self-awareness. Community sport being ‘*relatively*’ unconditioned means that social rules (boundaries) should exist to maintain a sense of safety for participants. A participant’s behavior might not be accepted, though he or she will not be rejected as a person. An important facilitating factor is the presence of a coach who knows his or her participants, and is familiar with the social vulnerabilities experienced by the participants. Helpful as well (for connecting) is the coach having a similar socioeconomic background, as such being a role model for participants. When coach or peers have some life experiences in common, it may reduce feelings of loneliness, help put participants’ problems into perspective or stimulate participants in finding the strength to improve their own situation. The main mechanisms identified are assurance, recognition and acceptance. Participants feel reassured by the fact that their coach has knowledge and understanding of participants’ living environment; they feel recognized by their coach, peers and society; and they feel understood and accepted as a person, regardless of their sports skills or social difficulties.

### Improved self-efficacy through motivational coaching (CMO 2)

*If participants experience a safe space wherein they are stimulated to take initiative and to learn by experience (C), then they enhance their self-awareness (iO), perceived self-efficacy (IO), and self-esteem (IO), because they build up self-acceptance and appreciation through success experiences (M).*

Participants being coached positively are reinforced in what they do well regarding their role in the team or regarding their sportive capacities, and therefore become increasingly aware of their own realizations and successes. Positively coached participants feel socially accepted and build up positive self-esteem. Emphasizing what participants do well allows them to identify themselves in a positive way (e.g. a player, team leader, responsible for the training gear…) as opposed to seeing themselves as, e.g., ‘the homeless one’, ‘the one who got expelled from school’ or ‘the one with the mental problems’.

### Sense of belonging and self-esteem through constructive group dynamics (CMO3)

***If****community sport provide participants the opportunity to get to know one another and to connect (C),****then****participants perceive a sense of belonging (IO) and improved self-esteem (IO)****because****they feel recognized and acknowledged in their role and in themselves (M).*

Participants with a background of vulnerability feel part of a group, a bigger entity; they feel noticed and known (‘*someone remembers your name*’) by peers and coaches. As such, participants identify themselves more positively, and feel no longer marginalized. Facilitators are to train in the same outfit (wearing clean and professional sportswear does not only improve feelings of belonging but also one’s self-esteem) and the team being linked to and recognized by a Premier League team (e.g. being invited on the field before a match, being on a picture with the Premier League players, wearing matching jerseys, having a trainer from the Premier League team) and pursuing a common goal. Factors that hinder a sense of belonging are: a focus on competition, possibly causing a drop-out of participants with poor physical or sport-technical skills, being often the most vulnerable persons of the target group.

### Mentoring participants in personal health goal-setting (CMO 4)

***If****a health and physical activity promoting climate exists in which desired behavior is visible and attractive (C), participants are provided opportunities to learn by experience, to become knowledgeable and self-aware and to increase self-efficacy (C),****then****participants become motivated to set (realizable) personal health goals (DO)****because****they know why and how to take actions towards self-care and healthy living, and are engaged to do so (M).*

Community sport coaches and peer experts serve as a role model regarding the link between healthy living, wellbeing and personal development facilitating social inclusion. They provide participants with access to information with regards to healthy behavior and how to make positive health choices. Especially peer experts, who have encountered similar challenges, may set a strong and inspiring example. Participants build up success experiences through reflection and are motivated to shift their physical and mental boundaries. Increases in self-efficacy with regard to physical activity may promote and sustain physical activity levels, possibly outside community sport.

Table 1 gives an overview of some examples of verbatim used for each of the CMO configurations, as well as examples of facilitating context factors for the concerned mechanism to be triggered.

### Program theory

Figure 1 presents a visual depiction of a program theory describing key mechanisms and important context factors in generating positive outcomes on health and wellbeing of socially vulnerable persons participating in community sport.

In Fig. 1, the distal outcomes of community sport programs are presented as the tips of an iceberg. The mechanisms – the core of the iceberg – are not visible and have to be revealed through deep realist inquiry. In the water surrounding the iceberg is the context, both facilitating (+) and limiting (−) conditions influencing (molding, triggering, eroding) the underlying latent mechanisms. Three initial outcomes (iO) may be among the immediate results of some easily manifested mechanisms and may be a prerequisite for the intermediate outcomes: 1) a sense of safety within the environment and interpersonally while performing community sport; 2) self-awareness about one’s own behavior and knowledge about exercise and health related behavior; and 3) mental space (i.e., (temporary) acceptance of oneself and one’s situation and openness to a community sport environment). Three intermediate outcomes (IO) of community sport have the potential to impact motivation to perform and maintain healthy behavior: 1) a sense of belonging (i.e., feeling related to the group and coaches while performing community sport; 2) a positive self-esteem (i.e., a sense of autonomy to be oneself, also while performing physical exercise and healthy behavior); 3) perceived self-efficacy (i.e., a sense of competence that one can perform and maintain physical exercise and undertake actions towards healthy behavior, and skills demonstrating the ability to be physically active and set health goals). Motivation to perform and maintain sports and healthy behavior is believed to be related to the actual behavior, and to better health and wellbeing on the long term (cf. discussion for existing theories supporting this association). There appears to be a relative sequencing to the mechanisms (M). An environment perceived by participants as a safe and trustworthy place (M1), where they feel accepted and can be one selves, is prior and generates the preconditions for keeping participants ‘in’, for motivating them to continue to sport and grow, and as such be exposed to positive coaching (M2) and constructive group dynamics (M3). Enabling personal health goal-setting (M4), on the other hand, appears to be one of the later mechanism to be triggered, since the context for enabling goal-setting needs to be sufficiently safe and mature: trusting relationships between coach and participant, and among participants; presence of role models; development of self-esteem and self-efficacy and constructive group dynamics are necessary conditions.

## Discussion

The study results suggest that community sport activities may contribute to health via an increased sense of belonging, positive self-esteem and perceived self-efficacy of socially vulnerable groups through mechanisms of motivational coaching and constructive group dynamics, including role modelling among peers. Sustainable behavior change is preceded by a long and winding road of personal development, it is a long-term process requiring time-demanding interactions and certain necessary conditions to be in place, among which a safe and trustworthy environment, coaches familiar with the meaning and implications of social exclusion, and strong partnerships between community sport organizations and other stakeholders.

In this process, community sport works as a soil improver; it prepares the necessary conditions for the personal growth of socially vulnerable individuals. Community sport activities are usually organized in a safe and trustworthy climate in which participants can be themselves, are allowed to make mistakes, feel accepted for who they are, and are encouraged to take initiative and responsibility – all important conditions for building success experiences. A safe climate in community sport also means that participants know what is expected from them, that they are offered structure and predictability (routine), and that norms and values adhered to in the group are clear [42, 43]. In psychologically safe environments, people believe that they will not be punished or thought less of when making a mistake of when asking for help, which fosters the confidence to take the risks associated with learning (i.e. the risk of being seen as ignorant, incompetent or negative), thereby gaining from the associated benefits of learning [44]. Our study showed that psychological safety fosters the participants’ ability to drop the – often heavy – emotional backpack and to be temporarily dismissed of responsibilities. This brings the necessary tranquility and what we have named in our study ‘mental space’ for participants of community sport to work on oneself, especially when experiencing the organized activities as fun and unconditioned. Several other studies confirm the importance of psychologically safe spaces in community sport [45, 46].

Another finding highlighted in our results is the importance of role models [47, 48], in community sport projects potentially embodied by coaches, professional sport players or peer experts. Especially the latter seems to be able to set a powerful example. In community sport activities, a peer expert is for example a long-term participant of the project who has gone a long and successful path of personal development and who gradually grew into a role as ‘elder brother or sister’ or who took on some responsibility within the project. Peer experts make caring for oneself and one’s health visible, valued and attractive, which increases awareness of other participants on why and how to live healthy. In a meta-review studying the effects of interventions on self-efficacy, physical activity self-efficacy is reported to be significantly higher when vicarious experience (i.e. seeing a similar other perform the concerned behavior) is included in the intervention [49].

When conditions of psychological safety, fun and mental space are fulfilled, participants in our study feel motivated to keep on participating after a first experience, as such advantaging of the ability to form meaningful relations with other participants and coach(es) and to make sense of their free time [50, 51]. Motivational coaching and positive group dynamics then become key mechanisms, encouraging participants to build success experiences [19, 52]. Coaches (herein followed by participants copying the coaches’ attitude) focus on what goes well, not on what goes wrong; the process is prior to the result. While ‘social persuasion’ [40] (i.e. encouragement and compliments) used as a stand-alone technique has been reported to have a weak impact on self-efficacy beliefs [49], our study results demonstrate that, in combination with other behavior change techniques, it may have an impact. Socially vulnerable persons seem to be profoundly touched by it, possibly because most of them are used to dealing with rejection, prejudgments and failure experiences [12, 20, 53]. Moreover, to have a role and a place in a group, to be part of a bigger whole and to be connected with others, gives people the feeling they have the right to be [51]. It increases participants’ self-confidence, perceived self-efficacy and sense of belonging, which appear in the study data as important building blocks for motivation to change one’s behavior. These elements are also key in the self-determination theory [41] and in the social cognitive theory [54]. A person who is motivated to take his health in own hands and to set his or her own goals, is more likely to start off on a road to sustainable behavior change [55].

Although psychological theories such as the theory of planned behavior [55], the social cognitive theory [40] and the self-determination theory [41] have been useful in explaining the associations between the sensitizing concepts and the links between C, M and O in our program theory, when not integrated in a more contextualized approach, they fall short in the attention for pathways by which social environmental phenomena affect cognitive and biologic regulatory processes [56]. Moreover, the rather individualistically oriented behavior change models may unintentionally imply that individuals are personally responsible. Especially from a public health point of view, more attention is needed for the context in which behavior change takes place, or better, can take place [56, 57]. That is why, in complement to the theories referred to above, we used the Capability-Opportunity-Motivation-Behavior (COM-B) framework [57] to link different concepts in our program theory, and to give a proper place to context factors. ‘Capability’ (**C**OM-B), referring to the individual’s psychological and physical capacity (including knowledge and skills) to engage in the concerned behavior, is represented in our PT by the initial (iO) and intermediate (IO) outcomes, mainly generated by the first three mechanisms (experiencing a safe climate; being positively coached; being part of constructive group dynamics). ‘Motivation’ (CO**M**-B) includes all brain processes that energize and direct behavior, inclusive of habitual processes, emotional responding and analytical decision-making. ‘Opportunity’ (C**O**M-B), representing the factors external to the individual that make the behavior possible or prompt it, equals the context in our PT. Both opportunity and capability may influence motivation, and all three of them (COM) can alter a behavior (B), just like behavior can alter capability, opportunity and motivation [57].

### Strengths, challenges and future research opportunities

One of this study’s main strengths, namely the program theory being partly grounded in data and not solely the result of creating hypotheses, has generated some challenges as well. Theory from case studies is complex theory. Creating rich and contextualized theories comes with the risk of drifting away from parsimony and clarity [58]. We tried to mitigate this challenge by several attempts to visualize the program theory (hence simplifying it, with the aim to enhance clarity on the links between the different components); by trying to bring a certain chronology in the program mechanisms; and by providing concrete examples (e.g. Table 1) linking data to concepts of the program theory. Also, our program theory reflects the idea that context elements at micro-level (safe environment, volunteering opportunities, role models…) indeed play a huge role as catalyzer for key mechanisms. However, the “upstream” social determinants of health, such as social disadvantage, risk exposure and social inequities play a fundamental role as well [59, 60]. Context elements at meso (organization, network, partnerships, local politics…) and macro level (policy, law and regulation…) may trigger or impede important context elements at micro level. Due to a multitude of data, we focused in the first research loop of our study on the mechanisms closest to the ‘reasoning’ of the target group [61]. However, more attention is needed for the cascade of context factors at structural (political and societal) level allowing (or impeding) these mechanisms. In further research loops, this can be altered. Lastly, in this first research phase (‘research loop’, as we prefer), more community sport project coordinators, coaches and social partners have been questioned then participants. This influences the identified mechanisms and contextual factors that are considered to be important. Since our program theory will be subject to further testing and refinement in following realist research loops, it is recommended that we then shift the focus to the participants’ reflections on this theory.

Theory-building from cases comes with many advantages as well, such as the likelihood of developing novel, testable and empirically valid theory that closely mirrors reality [58]. In order to ensure rigor in this qualitative study, we have used strategies of prolonged engagement, persistent observation and rich, thick data (three related strategies, implemented through an intense period of participative and non-participative observation, followed by interviews and focus groups only after a relation of trust had been established); negative case analysis (focused on the identification of context elements explaining why the outcome differed for that particular person or project activity); peer review debriefing; member checking (both in later interviews and focus groups); and triangulation [62, 63]. Our realist yet grounded theory-building approach allowed enhanced data validity and reliability in at least two ways [62, 64]. First, data were collected and analyzed in practice, in real-life settings. Since controlling the variables is not possible when studying complex social problems, it is important to know as much as possible about the variable in which the supposed key mechanisms function. Therefore, keen documentation of the context in which the mechanism is trigged, is required, and this preferably repeated in differing contexts and circumstances. Selection of the cases and the interviewees of interest to these cases has been done with respect to this principle. Second, although the study data were grounded in practice, analyzing them was a process of constant cross-pollination, both because of the transdisciplinary approach of the project (bringing together practitioners, academics and policy makers) and because of the fact that social scientists are always in contact with and influenced by existing theory, even when not aware of it [39]. At the one hand, the developed CMO configurations and the way they have been linked together confirm what various ‘grand’ theory has claimed before, which reinforced the reliability of the study data and oriented the shaping of program theory. At the other hand, the constant process of checking and discussion of findings with key stakeholders and social users of the CATCH project (all community sport organizations involved in or informed by the project), brought the analysis to a widely carried consensus.

### Study implications and recommendations for policy makers and practitioners

This study has contributed to the identification of facilitating conditions for community sport to play a health-promoting role for socially vulnerable groups (cf. Table 1), allowing program developers to consider essential working ingredients and contextual boundaries in setting-up successful health promotion initiatives. The study is also inspirational for developers and policy makers as it allows considering intermediate outcomes while evaluating programs, and interpreting (a due absence of) effects in light of mechanisms and conditions to be installed. We highlight some of the main key messages. First, since the community sport coach essentially acts as a change agent, an accurate ‘casting’ and ‘directing’ of community sport coaches is quintessential. It is recommended that community sport organizations map the different profiles available among the project’s human resources and evaluate the potential need for training in issues related to social vulnerability, personal development through sport, motivational coaching techniques and group dynamics. This enhances the capacity of coaches involved in the program to shape the context as such that necessary conditions are met for triggering the key mechanisms of community sport. Second, our data suggested the asset of involving peer experts in sport health programs. Therefore, we recommend efforts are made for identifying the right conditions for peer experts to take on a role in helping others to become more socially included, and consequently, for providing peer experts with opportunities to play this role in a safe and supported setting. Third, a strong link, excellent communication and a shared agenda with partner organizations are paramount to the set-up of effective sport health programs. Examples of relevant actors include the Social Welfare Council, job integration services and organizations working on prevention and health promotion. Fourth, structural project collaboration, sharing of material and human resources and shared monitoring and evaluation systems may significantly enhance the efficacy of community sport organizations. A strongly organized community sport network may also be an opportunity to bundle different short term project funding into a more substantial and stable project fund, allowing training and retention of community sport coaches as change agents and a long-term follow-up of project participants.

## Conclusion

Community sport can be a powerful lever for health promotion when certain conditions are met. A safe and trustworthy climate in which community sport participants can be themselves and learn by experience and from others, is the basis from which community sport coaches depart to assist socially vulnerable persons in setting and pursuing personal health goals, and to contribute to the participants’ resilience building trajectory. Although, for example, a decrease in use of tobacco, alcohol and drugs could be observed in some participants, loyal to the program, participating in community sport activities is rarely directly affecting people’s physical condition and health indicators. Participating in community sport activities makes socially vulnerable people feel better due to an increased self-esteem, self-efficacy and motivation to set and pursue health-related goals, resulting from processes of experiential learning among peers, incremental responsibility-taking and reflexivity. These processes, and the right context factors for these processes to occur, are mainly triggered and reinforced (or limited) by the ways in which the coach interacts with the participants and coaches the group. Therefore, this study stresses the need for reflection on community sport coaches’ required profile and skills set in order to be able to improve the soil and shape the necessary conditions for community sport to become a lever for health promotion.

## Supplementary information

**Additional file 1.** Overview of data collections.

**Additional file 2.** Initial Program Theory - CATCH Health promotion.

## Data Availability

The datasets generated and/or analyzed during the current study are not publicly available due to the unavailability of English translations for all of the transcripts, but are available from the corresponding author on reasonable request.
