# Supplementary material for: How community sport programs may improve the health of vulnerable population groups: a program theory
Source: Int J Equity Health. 2020 May 24;19:74. doi: 10.1186/s12939-020-01177-5 (PMC7245920; doi:10.1186/s12939-020-01177-5)
Supplement: Supplementary file 1 — Additional file 1. Overview of data collections. [file 12939_2020_1177_MOESM1_ESM.docx]

***Additional file 1: Overview of data collections***


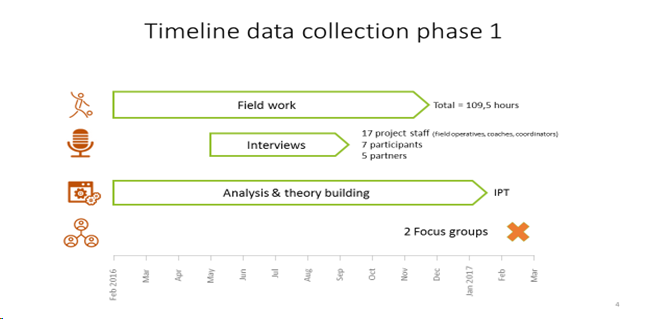


**Interviews**

| **ID** | **Province** | **Practice** | **Role** |
| --- | --- | --- | --- |
| R1 | Vlaams-Brabant | BLe | Field operative |
| R2 | Oost-Vaanderen | SCoG | Social partner’s staff member |
| R3 | Oost-Vlaanderen | GP | Staff member |
| R4 | Limburg | LHB | Field operative |
| R5 | Limburg | LHB | Field operative |
| R6 | Vlaams-Brabant | BLe | Staff member |
| R7 | Oost-Vlaanderen | SGN | Social partner’s staff member |
| R8 | Vlaams-Brabant | BLe | Field operative |
| R9 | Vlaams-Brabant | BLe | Field operative |
| R10 | Oost-Vlaanderen | VJ | Staff member |
| R11 | Oost-Vlaanderen | SWCoG | Social partner’s staff member |
| R12 | Oost-Vlaanderen | OS | Social partner’s field operative |
| R13 | Vlaams-Brabant | LSS | Staff member |
| R14 | Oost-Vlaanderen | GP | Field operative |
| R15 | Oost-Vlaanderen | VJ | Staff member |
| R16 | Oost-Vlaanderen | LSS | Field operative |
| R17 | Oost-Vlaanderen | VJ | Field operative |
| R18 | Oost-Vlaanderen | VJ | Field operative |
| R19 | Vlaams-Brabant | LSS | Field operative |
| R20 | Vlaams-Brabant | SWCoL | Social partner’s staff member |
| R21 | Limburg | LHB | Field operative |
| R22 | Oost-Vlaanderen | VJ | Field operative |
| R23 | Vlaams-Brabant | LSS | Participant |
| R24 | Oost-Vlaanderen | GP | Participant |
| R25 | Oost-Vlaanderen | GP | Participant |
| R26 | Oost-Vlaanderen | GP | Participant |
| R27 | Oost-Vlaanderen | GP | Participant |
| R28 | Oost-Vlaanderen | GP | Participant |
| R29 | Limburg | LHB | Participant |

**Focus group participants**

| **Respondents ID FG1** | **Province** | **Practice** | **Role** |
| --- | --- | --- | --- |
| FG1a | Antwerpen | CoL | Field operative |
| FG1b | Antwerpen | CoL | Field operative |
| FG1c | Limburg | YCoH | Field operative |
| FG1d | Limburg | YCoH | Field operative |
| FG1e | Oost-Vlaanderen | BRo | Field operative |
| FG1f | Vlaams-Brabant | YCoL | Field operative |
| **Respondents ID FG2** | **Province** | **Practice** | **Role** |
| FG2a | Oost-Vlaanderen | VJ | Field operative |
| FG2b | Oost-Vlaanderen | VJ | Staff member |
| FG2c | Oost-Vlaanderen | SNG | Field operative |
| FG2d | Oost-Vlaanderen | SG | Staff member |
| FG2e | Oost-Vlaanderen | WCG | Field operative |
| FG2f | West-Vlaanderen | BRoe | Field operative |
| FG2g | West-Vlaanderen | VA | Field operative |
