# Supplementary material for: How community sport programs may improve the health of vulnerable population groups: a program theory
Source: Int J Equity Health. 2020 May 24;19:74. doi: 10.1186/s12939-020-01177-5 (PMC7245920; doi:10.1186/s12939-020-01177-5)
Supplement: Supplementary file 2 — Additional file 2. Initial Program Theory - CATCH Health promotion. [file 12939_2020_1177_MOESM2_ESM.docx]

***Additional file 2: Initial Program Theory – CATCH health promotion***

| **CONTEXT** | **OFFER** | **MECHANISM** | **OUTCOME** | **IMPACT** |
| --- | --- | --- | --- | --- |
| Support for accessible offer of leisure activities | Free  Unconditional  Flexible  Outreaching | Youth experience less barriers to leisure offer. | Starting to move | Physical health |
| Collaboration with sport clubs | Initiation courses | Youth learn about leisure offer. | Keep on moving |  |
| Time investment of the attendants | Guiding youth to sport clubs | Youth experience less barriers to sport clubs. |  |  |
|  | Coaching of sport clubs | Sport clubs become aware of barriers. |  |  |
| Social-pedagogical attendants | Group activities | Youth have fun in group. | Group feelings | Mental health |
|  | Focused training and exercises |  |  |  |
|  | Non-competitive setting | Youth experience a sense of belonging. |  |  |
| Collaboration with partners | Positive coaching | Youth have success experiences | Self-confidence |  |
